# Intraoperative Transcranial Motor-Evoked Potential Monitoring During Head and Neck Surgeries: A Case of Cervical Vagus Nerve Schwannoma With Laryngeal Paralysis

**DOI:** 10.7759/cureus.30526

**Published:** 2022-10-20

**Authors:** Akihisa Tanaka, Hirokazu Uemura, Tsunenori Takatani, Masahiko Kawaguchi, Hironobu Hayashi, Tadashi Kitahara

**Affiliations:** 1 Otolaryngology-Head and Neck Surgery, Nara Medical University, Kashihara, JPN; 2 Otolaryngology-Head and Neck Surgery, Bellland General Hospital, Sakai, JPN; 3 Anesthesiology, Nara Medical University, Kashihara, JPN

**Keywords:** vagus nerve schwannoma, transcranial motor-evoked potential, intraoperative neuromonitoring, head and neck surgery, enucleation

## Abstract

Intraoperative transcranial motor-evoked potential (TcMEP) monitoring can effectively prevent neurological complications by enabling the evaluation of neurological deficits in all pathways from the motor cortex to the periphery. However, studies regarding its applicability in head and neck surgery are insufficient. This case report discusses a patient who was intraoperatively diagnosed with a right cervical vagus nerve schwannoma previously at another hospital. The patient then developed right laryngeal paralysis after the surgery without neuromonitoring. No significant recovery of the paralysis was observed, and after eight months of being referred to our institution, the patient opted for surgical retreatment following tumor growth and accompanying symptoms such as cervical swelling and discomfort. The patient was examined to evaluate the nerve damage in his previous surgery TcMEP monitoring as well as direct stimulation (DS). The right vagus nerve (RVN) showed no response on TcMEP monitoring throughout the surgery despite a significant response to DS at the tumor site. These findings suggest that the RVN had been damaged medial to the tumor site, and the damage occurred because of traction and ischemia during the previous surgery. Thus, contrary to our belief, medial nerve damage may be present even when local and peripheral nerve preservation is observed through peripheral neuromonitoring. This suggests that DS alone during neuromonitoring in head and neck surgery is insufficient. A multimodal evaluation approach, including TcMEP monitoring, is effective in not only preventing neurological complications but also in evaluating neurological deficits in all pathways from the motor cortex to the periphery.

## Introduction

The vagus nerve (VN) provides motor innervation to the larynx, including the vocal cord. VN damage may result in hoarseness or dysphagia and should be avoided during surgeries such as cervical neck dissection and thyroidectomy. In these surgeries, intraoperative neuromonitoring is conventionally performed by direct stimulation (DS) to the nerve.

Intraoperative transcranial motor-evoked potential (TcMEP) monitoring is an effective method of preventing neurological complications during brain and spinal surgeries [1,2], which can help evaluate the effects of intraoperative traction and ischemia on nerve roots and the central nervous system as well as local nerve damage [3]. A schwannoma is a benign nerve sheath tumor with an exceptionally high risk of intraoperative neurological complications; it is usually treated by enucleation [4]. The utility of intraoperative TcMEP for peripheral nerve schwannoma has been reported in other surgical specialties [2]. However, its use in head and neck surgeries has not been fully established.

Here, we present a case of cervical VN schwannoma with laryngeal paralysis that was evaluated by TcMEP monitoring in addition to DS.

## Case presentation

**Figure 1:**
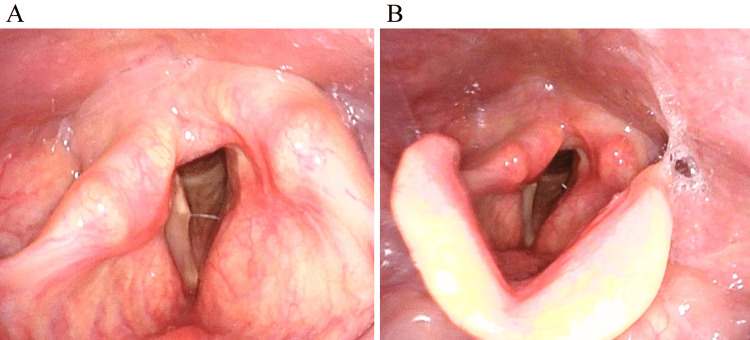
Endoscopic laryngeal findings. A: Before enucleation. B: After enucleation. The right vocal cord is fixed in a paramedial position on inspiration before and after enucleation.

A 42-year-old man referred to our institution presented with hoarseness and a right cervical mass. Right vocal cord palsy and right laryngeal paralysis were observed endoscopically although no dysphagia or dyspnea was observed (Figure 1, Panel A).

Moreover, the patient had already undergone surgical resection of the mass that could either have been a cyst or lymphadenopathy, but without neuromonitoring. However, it was intraoperatively identified as a cervical VN schwannoma. The surgery was completed without cutting the nerve sheath or excising the tumor, and the hoarseness occurred immediately post-surgery.

**Figure 2:**
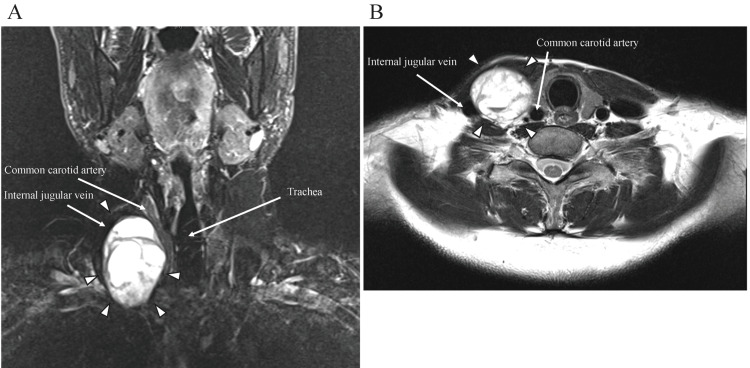
Results of magnetic resonance imaging. A: Short TI inversion recovery. B: T2-weighted. A well-defined lobulated heterogeneous mass with a split fat sign is located between the internal jugular vein and the common carotid artery (the tumor is shown surrounded by arrowheads).

The patient initially declined any further surgical intervention, hoping that the paralysis would recover, but visited regularly for follow-up. On his eighth-month check-up, magnetic resonance imaging revealed a 10-mm tumor growth (Figure 2), accompanied by right cervical swelling and discomfort.

Subsequently, he requested surgery to resect the tumor with neuromonitoring to evaluate the conduction of his right VN. Therefore, enucleation was performed using intraoperative TcMEP monitoring in conjunction with DS.

TcMEP was evaluated using the Neuromaster neurophysiologic monitoring system (Nihon Kohden Ltd., Tokyo, Japan). Corkscrew electrodes were placed on the scalp at C3 and C4, following the international 10-20 system. Four single-pulse trains with an interstimulus interval of 2 ms were used for transcranial stimulation. The transcranial stimulation intensity was set 20% higher than the threshold level to ensure that MEPs with amplitudes of at least 50 µV could be stably detected during surgery. The response to DS at intensities of 0.5-1.0 mA was also recorded. The responses of the recurrent laryngeal nerve to transcranial stimulation and DS were recorded using the nerve integrity monitor electromyography (NIM EMG) endotracheal tube (NIM-Response 3.0, Medtronic Japan Co., Ltd, Tokyo, Japan).

General anesthesia was maintained with propofol (2.8-4 μg/mL; using a target-controlled infusion pump; TE-371, Terumo Corporation, Tokyo, Japan) and remifentanil (0.26-0.31 μg/kg/minute). Rocuronium (50 mg) was also administered for muscle relaxation before tracheal intubation. The anesthesia depth was monitored using the bispectral index.

**Figure 3:**
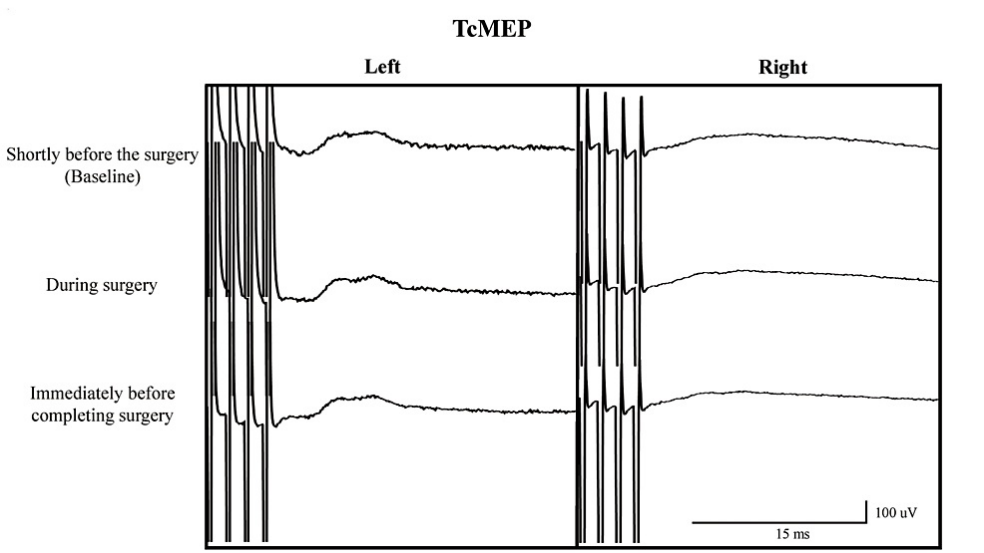
Waveform of the transcranial motor-evoked potential. The right vagus nerve shows no response to transcranial stimulation throughout the surgery. The left vagus nerve shows significant responses. TcMEP: transcranial motor-evoked potential

The baseline TcMEP was recorded shortly before the surgery. The right VN (RVN) had no significant response to transcranial stimulation, but the left VN (LVN) showed a significant response (Figure 3).

**Figure 4:**
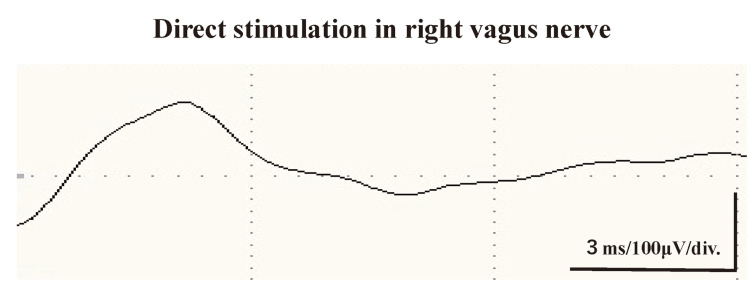
Waveform of the direct stimulation to the right vagus nerve. A significant response is identified.

On DS of the tumor through a collar incision in the anterior neck, significant diffused responses were observed on the tumor (Figure 4).

**Figure 5:**
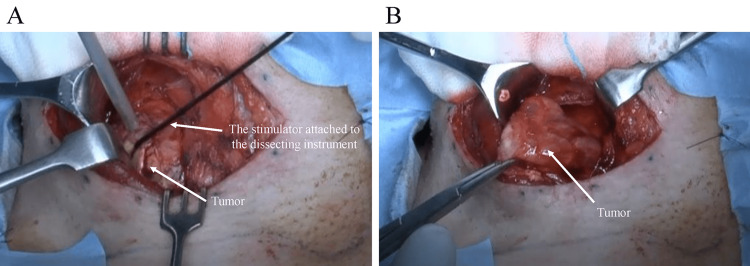
Intraoperative photographs. A: The tumor is enucleated using the stimulator attached to the dissecting instrument. B: The tumor is enucleated and resected.

Similar DS responses were detected on proximal and distal VN fibers of the tumor too as far as the cervical surgical field was visible. After making a longitudinal incision in the nerve sheath, the tumor was enucleated step-by-step along the tumor capsule (Figure 5).

Although TcMEP monitoring showed no responses in the RVN throughout the surgery, the LVN was significantly responsive. The neural response was confirmed by DS throughout the surgery.

The patient was histologically diagnosed with a benign cervical VN schwannoma measuring 50 × 33 × 27 mm. At the final follow-up one year later, the patient was stable, although hoarseness and complete right laryngeal paralysis persisted (Figure 1, Panel B).

## Discussion

Neurological deficit is a critical complication of head and neck surgeries, which can effectively be prevented by intraoperative neuromonitoring by DS and NIM EMG endotracheal tube. TcMEP monitoring can confirm neurological deficits by detecting peripheral neural responses to motor cortex stimulation [1,2]. However, reports on its applicability for head and neck surgeries are limited.

The present case discusses post-surgical right laryngeal paralysis without any direct cutting-induced injury to the tumor. The patient originally did not have any laryngeal paralysis or hoarseness. Transient laryngeal paralysis, which is not a direct result of incision-induced injury to the nerve after VN schwannoma enucleation and thyroid surgeries, can recover within several months. Thus, the patient was followed up without any additional treatment, hoping for functional recovery after neurapraxia. However, the patient requested surgical treatment of recurrent tumor growth and evaluation of accompanying neuropathy. Therefore, enucleation was performed with intraoperative TcMEP monitoring and DS. There was no baseline response from the RVN. The reliability of TcMEP monitoring may be affected by factors such as anesthesia, body temperature, and blood pressure [5]. However, they were considered to be controlled, as demonstrated by the responses from the LVN. The results indicated RVN damage. Initially, it was assumed that the damage had occurred at the tumor site. However, the response to DS was confirmed at the surgical site. These results suggest that the RVN had been damaged medial to the tumor site, although the nerve remained responsive at the periphery. They also suggest that the RVN paralysis occurred because of traction and ischemia, as opposed to local nerve damage, during the previous surgery. TcMEP monitoring prevents intraoperative nerve damage. This case of preoperative nerve paralysis highlights its importance in evaluating both central and peripheral nerve pathways. TcMEP monitoring is expected to be useful in head and neck surgeries involving monitorable motor nerves, as in this case, although it requires more effort to maintain its reliability than DS.

## Conclusions

This case showed that medial nerve damage cannot be ruled out in head and neck surgery even if DS neuromonitoring does not detect any local or peripheral nerve damage at the surgical site. Most importantly, a multimodal evaluation, employing both DS and the TcMEP monitoring approach, is effective in preventing neurological complications and evaluating neurological deficits in all pathways from the motor cortex to the periphery.
